# Generation of two induced pluripotent stem cell lines from patients with cardiac amyloidosis carrying heterozygous transthyretin (TTR) mutation

**DOI:** 10.1016/j.scr.2023.103215

**Published:** 2023-09-27

**Authors:** Bernardo Bonilauri, Hye Sook Shin, Min Htet, Christopher D. Yan, Ronald M. Witteles, Karim Sallam, Joseph C. Wu

**Affiliations:** a Stanford Cardiovascular Institute, Stanford University School of Medicine, Stanford, CA 94305, USA; b Department of Medicine, Division of Cardiology, Stanford University School of Medicine, Stanford, CA 94305, USA; c Greenstone Biosciences, Palo Alto, CA 94305, USA

**Keywords:** iPSC, Stem cells, Pluripotency, TTR, Transthyretin, Amyloid, Cardiac amyloidosis

## Abstract

Specific mutations in the TTR gene are responsible for the development of variant (hereditary) ATTR amyloidosis. Here, we generated two human induced pluripotent stem cell (iPSC) lines from patients diagnosed with Transthyretin Cardiac Amyloidosis (ATTR-CM) carrying heterozygous mutation in the TTR gene (i.e., p.Val30Met). The patient-derived iPSC lines showed expression of high levels of pluripotency markers, trilineage differentiation capacity, and normal karyotype. The generation of these iPSC lines represents a great tool for modeling patient-specific amyloidosis *in vitro*, allowing the investigation of the pathological mechanisms related to the disease in different cell types and tissues.

## 1. Resource utility

Human induced pluripotent stem cell (iPSC) technology represents a promising tool for use in precision medicine and drug discovery, as well as being an unlimited source to generate distinct cell types (e.g., cardiomyocytes, fibroblasts, neurons, and hepatocytes) for *in vitro* disease modeling (Giadone et al., 2018; Lau et al., 2019). Here, we generated two iPSC lines from male patients diagnosed with Transthyretin Cardiac Amyloidosis (ATTR-CM) carrying a heterozygous mutation (c.148G > A) in the transthyretin (*TTR*) gene (Table 1).

## 2. Resource details

Transthyretin Amyloidosis (ATTR) is a severe systemic and fatal disease caused by a misfolding and aggregation of the normal, non-mutated transthyretin protein (wild-type ATTR) or by a heterozygous mutation in the *TTR* gene (hereditary/variant ATTR) that promotes misfolding and aggregation. Usually, the clinical presentation of the disease includes polyneuropathy (ATTR-PN) and/or cardiomyopathy (ATTR-CM) caused by the deposition of these amyloid aggregates and fibrils into the target organs. Over 130 mutations in the *TTR* gene have been identified, most of them being pathogenic (Adams et al., 2019; Ruberg and Berk, 2012). Here, we described the generation of two iPSC lines derived from a 65 year-old Caucasian male patient (SCVIi066-A) and a 63 year-old Caucasian male patient (SCVIi067-A) diagnosed with transthyretin cardiac amyloidosis due to a heterozygous mutation in the TTR gene (c.148G>A encoding p.Val30Met; pathogenic variant). The mutation Val30Met (also known as V50M) is one of the most common mutations in the *TTR* gene and is endemic in some countries (e.g., Portugal and Sweden), presenting with clinical manifestations related to ATTR-PN with or without ATTR-CM (Adams et al., 2019). Cardiac manifestations of TTR-CA include a restrictive cardiomyopathy phenotype, reduced stroke volume, compromised cardiac output, atrial fibrillation, diastolic dysfunction, and cardiac fibrosis (Griffin et al., 2021). Therefore, the cell reprogramming approach provides an unlimited source for generating a plethora of cell types affected by the Transthyretin Amyloid disease, such as hepatocytes (the main source of TTR production), cardiomyocytes (target tissue of ATTR deposition) and peripheral nerves (target tissue of ATTR deposition), allowing *in vitro* disease modeling and drug screening assays (Giadone et al., 2018).

**Fig. 1.**
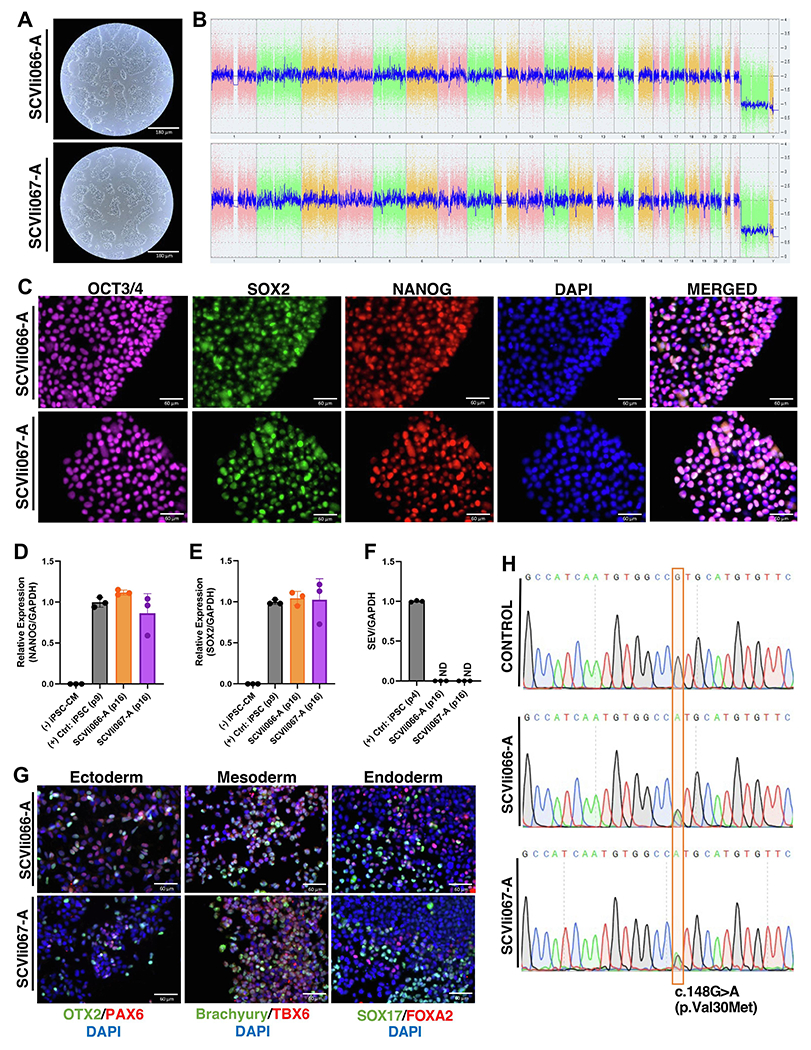
Characterization of cardiac amyloidosis patient-derived iPSC lines (SCVIi066-A and SCVIi067-A) with c.148G > A mutation in *TTR* gene.

Reprogramming of a patient’s peripheral blood mononuclear cells (PBMCs) to iPSCs was performed using Sendai virus containing the four Yamanaka factors (Oct3/4, Sox2, Klf4, and c-Myc) (see Material and Methods). iPSC clones (SCVIi066-A and SCVIi067-A) showed typical morphology (Fig. 1A) and normal karyotype (Fig. 1B). Immunofluorescence staining showed the expression of pluripotency markers OCT3/4, NANOG and SOX2 at the protein level (Fig. 1C). Quantitative analysis of gene expression of *NANOG* and *SOX2* was confirmed by reverse transcription-quantitative polymerase chain reaction (RT-qPCR) (Fig. 1D–E, respectively). Both genes presented mRNA levels as high as a control iPSC (i.e., healthy non-mutated cells) and significantly higher than iPSC-derived cardiomyocytes (iPSC-CMs) generated from the control line. Furthermore, expression of the non-integrative Sendai virus (SEV) was absent from both lines at passage 16 (Fig. 1F). The generated iPSC lines were able to successfully differentiate into all three germ layers (i.e., ectoderm, mesoderm, and endoderm) (Fig. 1G). The presence of heterozygous mutation (c.148G>A) was confirmed by Sanger sequencing and was absent in the control cell line (Fig. 1H). SCVIi066-A and SCVIi067-A lines were mycoplasma-negative (Supplementary Fig. 1A). Short tandem repeat (STR) analysis confirmed that both lines demonstrated overlapping profiles with corresponding somatic donor cells (submitted in archive with journal).

## 3. Materials and methods

### 3.1. Reprogramming

Peripheral blood mononuclear cells (PBMCs) were isolated from patient’s whole blood by Percoll^®^ density gradient separation medium (GE Healthcare #17089109) and purified by multiple washing using DPBS (Thermo Fisher Scientific #14190144). Briefly, PBMCs were cultured in StemPro^®^-34 SFM medium (Thermo Fisher Scientific #10639011) supplemented with 100 ng/mL SCF (Peprotech #300-07), 100 ng/mL FLT3 (Thermo Fisher Scientific #PHC9414), 20 ng/mL IL-3 (Peprotech #200-3), 20 ng/mL IL-6 (Thermo Fisher Scientific #PHC0063) and 20 ng/mL EPO (Thermo Fisher Scientific #PHC9631). The medium was changed every two days until cell culture stabilization. CytoTune^™^-iPSC 2.0 Sendai Reprogramming Kit (Thermo Fisher Scientific #A16517) was used to perform cell reprogramming according to the manufacturer’s instructions. Transduced cells were plated in a Matrigel-coated plate and cultured in StemPro^®^-34 medium. Medium was replaced every two days until day 7, when the medium was switched by supplemented StemMACS^™^ iPS-Brew XF medium (Miltenyi Biotec #130-104-368) until day 10–15 post-transduction when colonies appeared and were ready for clonal expansion. The selected colonies were expanded and cryopreserved for future experimental use.

### 3.2. Cell culture

Patient-derived iPSCs were cultured in StemMACS^™^ iPS-Brew XF medium (Miltenyi Biotec, #130-107-086 and #130-107-087) until cells reached 90 % of confluency. Once cells reached confluency, they were passaged using 0.5 mM EDTA (Invitrogen, #15575-038) and seeded again on Matrigel-coated plates (Corning, #356231). StemMACS^™^ iPS-Brew XF plus 10 μM of ROCK inhibitor Y-27632 (Selleck Chemicals #S1049) were used for culturing cells, with Y-27632 being removed after 24 h. Cells were maintained in a 37 °C incubator, with 5 % CO_2_.

### 3.3. Trilineage differentiation

Differentiation capacity into cells of the three germ layers was performed using the STEMdiff^™^ Definitive Endoderm Differentiation Kit (STEMCELL^™^ Technologies #05110) for endoderm differentiation. Ectoderm differentiation was induced with the Human Pluripotent Stem Cell Functional Identification Kit (R&D Systems #SC027B). Mesoderm differentiation was induced using RPMI media supplemented with 827-Minus Insulin (Gibco #11875-085 and #A18956-01) for 48 h.

### 3.4. Immunofluorescent staining

For qualitative analysis of pluripotency and trilineage differentiation, cells were fixed in 4 % paraformaldehyde (PFA) at room temperature (RT) for 10 min. Afterwards, cells were permeabilized using 50 μg/mL digitonin (Sigma Aldrich #D141) for 10 min, followed by a blocking step using a solution of 1 % of Bovine Serum Albumin (BSA) for 30 min at RT. Cells were incubated with primary antibodies (Table 2) overnight at 4 °C. Cells were washed three times and incubated for 30 min at RT with secondary antibodies (Table 2). Nuclei were stained with Molecular Probes NucBlue^™^ (Thermo Fisher Scientific #R37606) for 10 min at RT.

### 3.5. RT-qPCR

For quantitative analysis of pluripotency, total RNA from iPSCs was extracted and isolated using the Direct-zol^™^ RNA Miniprep Kit (ZYMO Research #3R2061). cDNA was generated using iScript^™^ cDNA Synthesis Kit (BioRad #1708891) according to the manufacturer’s instructions. *SOX2, NANOG* and non-integrative Sendai virus (SEV) were amplified using commercial primers (Table 2) and TaqMan^™^ Gene Expression Assay (Applied Biosystems^™^ #4444556).

### 3.6. Short tandem repeat (STR) analysis

Genomic DNA (gDNA) was isolated from iPSCs (passage number 20) and PBMCs using the DNeasy Blood & Tissue Kit (Qiagen #69504). STR analysis was performed using CLA Identifiler^™^ Direct PCR Amplification Kit (Thermo Fisher Scientific #A44661). Capillary electrophoresis was performed on ABI3130xl by the Stanford Protein Nucleic Acid (PAN) Facility.

### 3.7. Karyotyping

Approximately 2 × 10^6^ cells were collected from the iPSC lines at passage number 11 and analyzed using the KaryoStat^™^ assay (Thermo Fisher Scientific).

### 3.8. Sanger sequencing

PCR primers were designed to detect TTR mutations (Table 2) and used to amplify the genomic region of the gDNA using the KOD One PCR Master Mix (DiagnoCine, #KMM-101). The PCR reaction was performed using the following conditions: 94 °C 1 min, 94 °C 30 s; 62 °C 15 s, 68 °C 1 min for 35 cycles / 68 °C 5 min. Sanger sequencing was submitted to and performed by Azenta.

### 3.9. Mycoplasma detection

Mycoplasma contamination was evaluated using the MycoAlert^™^ Detection Kit (Lonza #LT07-318) following manufacturer’s instructions.

## Supplementary Material

Supplementary Material

## Figures and Tables

**Table 1:** 

Classification	Test	Result	Data
**Morphology**	Photography brightfield	Normal	Fig. 1A
**Genotype**	Karyotype (G-banding) and resolution	Karyostat^™^ Assay, resolution 1–2 Mb: Normal karyotype 46, XY.	Fig. 1B
**Phenotype**	Qualitative analysis: Immunofluorescence staining	Positive expression of pluripotency markers: Oct3/4, Nanog and Sox2	Fig. 1C
**Identity**	STR analysis	16 loci tested match well	Submitted in archive with journal
**Differentiation potential**	Directed differentiation	Positive IF staining of three germ layers markers	Fig. 1G
**list of recommended germ layer markers**	Expression of these markers has to be demonstrated at mRNA (RT-qPCR) or protein (IF) levels, at least 2 markers need to be shown per germ layer	Positive expression of germ layers markers: Ectoderm: *PAX6, OTX2* Endoderm: *SOX17, FOXA2* Mesoderm: *BRACHYURY, TBX6*	Fig. 1G
**Mutation analysis**	Sequencing	Heterozygous SCVIi066-A (c.148G > A) SCVIi067-A (c.148G > A)	Fig. 1H
**Microbiology and Virology**	Mycoplasma	Mycoplasma testing by luminescence: Negative	Supplementary Fig. 1A
**Donor screening (Optional)**	HIV 1 + 2 Hepatitis B, Hepatitis C	N/A	N/A
**Genotype additional info (Optional)**	Blood group genotyping HLA tissue typing	N/A	N/A

**Table 2:** Reagents details.

	Antibodies used for immunocytochemistry/flow-cytometry			
	Antibody	Dilution	Company Cat #	RRID

Pluripotency marker	Rabbit Anti-Nanog	1:200	Proteintech Cat #142951–1-AP	RRID: AB_1607719
Pluripotency marker	Mouse IgG2bκ Anti-Oct-3/4	1:200	Santa Cruz Biotechnology Cat #sc-5279	RRID: AB_628051
Pluripotency marker	Mouse IgG1κ Anti-Sox2	1:200	Santa Cruz Biotechnology Cat #sc-365823	RRID: AB_10842165
Ectoderm marker	Goat Anti-Otx2	1:200	R&D Systems Cat #963273	RRID: AB_2157172
Ectoderm marker	Rabbit Anti-Pax6	1:100	Thermo Fisher Scientific Cat #42–6600	RRID: AB_2533534
Endoderm marker	Goat Anti-Sox17	1:200	R&D Systems Cat #963121	RRID: AB_355060
Endoderm marker	Rabbit Anti-Foxa2	1:250	Thermo Fisher Scientific Cat #701698	RRID: AB_2576439
Mesoderm marker	Goat Anti-Brachyury	1:200	R&D Systems Cat #963427	RRID: AB_2200235
Mesoderm marker	Rabbit Anti-Tbx6	1:200	Thermo Fisher Scientific Cat #PA5-35102	BRID: AB_2552412
Secondary antibody	Alexa Fluor 488 Goat Anti-Mouse IgG1	1:1000	Thermo Fisher Scientific Cat #A-21121	RRID: AB_2535764
Secondary antibody	Alexa Fluor 488 Donkey Anti-Goat IgG (H + L)	1:1000	Thermo Fisher Scientific Cat #A-11055	RRID: AB_2534102
Secondary antibody	Alexa Fluor 555 Goat Anti-Rabbit IgG (H + L)	1:500	Thermo Fisher Scientific Cat #A-21428	RRID: AB_141784
Secondary antibody	Alexa Fluor 647 Goat Anti-Mouse IgG2b	1:250	Thermo Fisher Scientific Cat #A-21242	RRID: AB_2535811
	Primers			
	Target	Size of band	Forward/Reverse primer (5′-3′)	

Sendai virus Plasmids (qPCR)	Sendai virus genome	181	Mr04269880_mr
Genotyping	*TTR:* (c.148G > A) Heterozygous	980 bp	5′-TGGGTCTGGATGTAGTTCTGACA-3′ 5′-AGCTTTGGTGTTACCCAGggaca-3′	
House-keeping gene (qPCR)	*GAPDH*	471	Hs02786624_g1	
Pluripotency marker (qPCR)	*SOX2*	258	Hs04234836_s1	
Pluripotency marker (qPCR)	*NANOG*	327	Hs02387400_g1	

**Table:** Resource table

Unique stem cell line identifier	1. SCVIi066-A 2. SCVIi067-A
Alternative name(s) of stem cell line	1. N/A 2. N/A
Institution	Stanford Cardiovascular Institute, Stanford, CA, USA
Contact information of distribution	Joseph C. Wu; joewu@stanford.edu
Type of cell lines	Induced pluripotent stem cells (iPSCs)
Origin	Human
Additional origin info required	Age: 65 years old (SCVIi066-A) and 63 years old (SCVIi067-A). Sex: Male (both). Ethnicity: Caucasian (both).
Cell source	Peripheral blood mononuclear cells (PBMCs)
Clonality	Clonal
Method of reprogramming	Sendai virus vectors
Genetic Modification	Yes
Type of genetic modification	Spontaneous mutation
Evidence of reprogramming	RT-qPCR / Immunofluorescence
Associated disease	Transthyretin Amyloidosis
Gene/locus	TTR (18q12.1) SCVIi066-A: heterozygous TTR (c.148G>A) SCVIi067-A: heterozygous TTR (c.148G>A)
Date archived/stock date	SCVIi066-A (11/11/2019) SCVIi067-A (09/21/2022)
Cell line repository/biobank	https://hpscreg.eu/cell-line/SCVIi066-A https://hpscreg.eu/cell-line/SCVIi067-A
Ethical Approval	The generation of the line was approved by the Administrative Panel on Human Subjects Research (IRB) under IRB #29904 “Derivation of Human Induced Pluripotent Stem Cells (Biorepository)”.
