# Supplementary figures and images for: Generation of two induced pluripotent stem cell lines from patients with cardiac amyloidosis carrying heterozygous transthyretin (TTR) mutation
Source: Stem Cell Res. Author manuscript; Available in PMC 2024 Jan 27. (PMC10821799; doi:10.1016/j.scr.2023.103215)

**A**      Mycoplasma

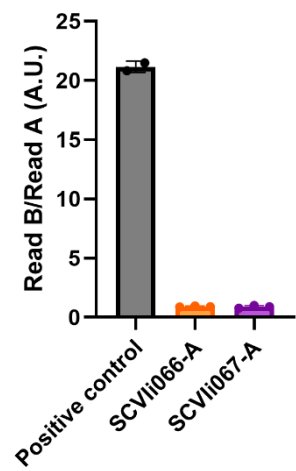

Supplement: Supplementary Material [file NIHMS1955706-supplement-Supplementary_Material.pdf]
